# Retraction: Inclined analysis of water diversion project supply chain profits in the occurrence of whole supply chain damage in undeveloped regions of China

**DOI:** 10.1371/journal.pone.0346947

**Published:** 2026-04-13

**Authors:** The PLOS One Editors

The *PLOS One* Editors retract this article [1] due to concerns about:

- Potential manipulation of the publication process.
- Peer review reliability.
- Insufficient detail of the specific datasets and sources of data used in this study to be reproducible.
- Citations that do not correspond to in-text citations, appear to be irrelevant to the cited statements, or cannot be retrieved.

SZA did not agree with the retraction. HW and ZM either did not respond directly or could not be reached.
